# Supplementary material for: Attitudes of blood and plasma donors towards biobanking for longitudinal health research
Source: PLoS One. 2025 Dec 18;20(12):e0338494. doi: 10.1371/journal.pone.0338494 (PMC12714250; doi:10.1371/journal.pone.0338494)
Supplement: S1 File — (DOCX) [file pone.0338494.s001.docx]

**Appendix – Survey**

Questionnaire Biobank

Start of Block: Consent form test subjects

**Research on attitudes towards the storage of bodily material**

| 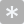 |
| --- |

**Required fields**

- I hereby consent to participating in this research on attitudes towards the storage of bodily material, conducted by the ‘Donor Studies’ research group at Sanquin. (1)
- I hereby confirm that I am 18 years of age or older. (2)
- I hereby confirm that I have read the information for test subjects and that I have had the opportunity to ask any additional questions. My questions have been answered to my satisfaction. I had sufficient time to decide whether or not I wanted to participate in this research. (3)
- I am aware that participating in this research is wholly voluntary. I agree to participate in this research. (4)
- I am aware that I can decide not to participate in the research at any time and that I can revoke my consent at any time, even after filling in the questionnaire. (5)
- I agree to allow Sanquin to store my anonymous data for a period of 10 years after the publication of the results of the research in a scientific journal. (6)
- I want to participate in this research. (7)

End of Block: Consent form test subjects

Start of Block: Demographic information

Did you know that after every blood donation a very small amount of blood is left over? Sanquin would like to put your donation to even better use by storing that small amount of residual blood for scientific research. We are curious to know what your attitudes and ideas are around the storage of material and data and the sharing of this information with other researchers. We ask you to please fill in this anonymous questionnaire, which will take about 5 to 10 minutes of your time. In doing so, you are not providing consent to having the residual blood from your donation be stored for scientific research. We are only interested in your opinion on this subject.

**1. How old are you?**

- Younger than 25 years old (1)
- 25-35 years old (2)
- 36-45 years old (3)
- 46-55 years old (4)
- 56-65 years old (5)
- Older than 65 years old (6)

**2. What is your gender?**

- Male (1)
- Female (2)
- Other, namely (3) ________________________________________________

**3. What is the highest level of education you have successfully completed with diploma?**

- None (1)
- Elementary school (2)
- Lower vocational education (VMBO, LBO: for example: LTS, LAS, LHNO, VBO, LEAO) (3)
- Secondary education (for example: ULO, MULO, MAVO, LAVO, VGLO) (4)
- Secondary vocational education (MBO: for example: MDGO, MAS, MTS, MEAO) (5)
- Higher secondary education (for example: HAVO, VWO, MMS, HBS, *atheneum*, *gymnasium*) (6)
- Higher vocational education (HBO: for example: HTS, HAS, HEAO, PABO) (7)
- University (8)

**4. In which province do you live?**

- Drenthe (1)
- Flevoland (2)
- Friesland (3)
- Gelderland (4)
- Groningen (5)
- Limburg (6)
- North Brabant (7)
- North Holland (8)
- Overijssel (9)
- South Holland (12)
- Utrecht (10)
- Zeeland (11)
- Other, namely (13) ________________________________________________

**5.** **Have you ever donated blood or plasma to the Sanquin blood bank?**

- Yes (1)
- No (2)
- I don’t know (3)

Display This Question:

If 5. Have you ever donated blood or plasma to the Sanquin blood bank? = Yes

**5b. How often did you donate blood to the Sanquin blood bank?**

Display This Question:

If 5. Have you ever donated blood or plasma to the Sanquin blood bank? = Yes

**5c. At which location do you generally make your donations?**

▼ Aalten (1) … Zwolle (129)

End of Block: Demographic information

Start of Block: Questions about the biobank

  **1. To what extent are you familiar with the term “biobank”?**

- I am familiar with it (1)
- I have heard of it (2)
- I am not sure (3)
- I have never heard of it (4)

| Page Break |  |
| --- | --- |

A biobank is a collection of bodily materials (blood, for example) and data that can be used to conduct scientific research on health and disease. A biobank is a kind of vault of information for researchers. General information on biobanks can be found at [www.biobanken.nl](http://www.biobanken.nl).

**Sanquin Biobank**

Every time a blood donation is made, a little bit of residual blood (about a teaspoon) remains in the ‘sample bag’ part of the collection bag. This material cannot be used to treat patients and is usually just disposed of. This residual blood could be put to good use in research on improving the blood supply, the health of donors, and improving public health and medical care. When you donate blood, you can give permission via the consent form for your residual blood to be used for research purposes. The new development with this biobank is that we would like to be able to store this residual blood for a longer period of time and use the samples multiple times for research purposes.

**2. Would you consent to the storage and use of residual blood in a biobank if you were to donate blood to Sanquin?**

- Definitely (1)
- Probably (2)
- I don’t know (3)
- Probably not (4)
- Definitely not (5)

**3. What are your considerations in terms of providing consent, or not, for the storage of residual blood in a biobank? (You may provide more than one answer)**

- I think it is wasteful if residual blood is simply disposed of (1)
- I think it is important to make a contribution to science (2)
- It is a small effort (3)
- Because I may be able to help others with this (4)
- Because I am not sure what a biobank is (5)
- Because I would first like to have more information on what exactly happens to the residual blood and
  data (6)
- Because I would first like to know how much time it will take (7)
- Because I have other doubts, namely (8) ________________________________________________
- Because it takes extra time (9)
- I am not sure what will be done with my blood (10)
- I am afraid that diseases or abnormalities will be detected (11)
- I have doubts about whether or not my personal data will be kept private (12)
- Other, namely (13) ________________________________________________
- None (14)

**4. Would you be willing to fill in questionnaires about your health and lifestyle if you were to participate in the biobank?**

- Definitely (1)
- Probably (2)
- I don’t know (3)
- Probably not (4)
- Definitely not (5)

**5. What are your considerations in terms of being willing, or not, to fill in questionnaires if you were to participate in the biobank? (You may provide more than one answer)**

- I think it is important to make a contribution to science (1)
- It is a small effort (2)
- Because I may be able to help others with this (3)
- Because I am unsure what the questionnaires entail (4)
- Because I have other doubts, namely (5) ________________________________________________
- Because it takes extra time (6)
- I am not sure what will be done with my data (7)
- I have doubts about whether or not my personal data will be kept private (8)
- I don’t like to share personal information with others (9)
- Other, namely (10) ________________________________________________
- None (11)

| Page Break |  |
| --- | --- |

Generally speaking, data collected for scientific research are kept for 15 years. In a biobank, data and bodily materials are often kept for even longer. This is done because new insights and research questions might arise in the future as a result of changes in knowledge, technology and public health. By storing the material for a longer period of time, these research questions can be answered using this material. We store data and bodily materials using a coding system, which means that all the information that we collect is only assigned with a numeric code. The key to the code is stored in a secure place at Sanquin. Whenever we make use of the data and bodily materials, we use only the numeric code.

**6. What would you consent to?**

- Bodily material and data may be stored for 30 years (1)
- Bodily material and data may be stored for 15 years (2)
- I would not consent at all (3)
- Other, namely (4) ________________________________________________

In the context of biobank research, it may be the case that your data and bodily material are also used by other researchers. These researchers may even work in a European country other than the Netherlands, or in a country outside of the European Union. While these countries may not share the same privacy laws as the European Union, it will always be guaranteed that your privacy is protected.

**7. Which of the following statements applies to you?**

- My bodily material and data may be shared with others (both in and outside Europe) as long as my privacy is protected (1)
- My bodily material and data may be shared with others (in the Netherlands) as long as my privacy is protected (2)
- My bodily material and data may not be shared with others (outside of Sanquin) (3)
- Other, namely (4) ________________________________________________

Organisations that may wish to make use of a biobank include hospitals or universities, as well as businesses that develop medical products (like medicines, vaccines or hospital equipment), and/or businesses that produce non-medical products (like nutritional supplements or health apps).

**8. What would you consent to? Biobank material and questionnaire data may be used by… (You may provide more than one answer)**

- Researchers that work at the blood bank, hospitals, universities, or other organisations that are active in the area of medical scientific research (1)
- Non-commercial businesses (2)
- Businesses, even if they are commercial enterprises (3)
- I would not give my consent at all (4)
- Other, namely (5) ________________________________________________

In order to establish a more comprehensive view of the health and background of biobank participants, researchers would like to make use of information from medical records from general practitioners, hospitals, and/or other institutions. These medical records can include information on hospital admissions, diagnoses, treatments and medication. Information from national registries and other sources may also be relevant to the biobank, like data from Statistics Netherlands (*Centraal Bureau voor de Statistiek*, CBS) for causes of death, information on forests and parks or fast-food chains in the area in which you live, or physical activity recorded with apps, among other things.

**9. What would you consent to? (You may provide more than one answer)**

- Biobank data may be linked to information from medical records (1)
- Biobank data may be linked to information from registries (like Statistics Netherlands) (2)
- Biobank data may be linked to information from other sources (like information on my residential area, related to stores, nature, air pollution, etc.) (3)
- Biobank data may be linked to information from apps or other online registries (like activity trackers, supermarket purchases, etc.) (4)
- Biobank data may not be linked to other sources of information (5)
- Other, namely (6) ________________________________________________

| Page Break |  |
| --- | --- |

Some illnesses are caused by parts of (genes) hereditary material (DNA) that increase the risk of particular diseases. Researchers would like to study the extent to which genes, lifestyle changes, and changes in blood values are related to the development of disease. This will help them to better understand the causes of disease. This information could be used in the future to detect a disease earlier, or to develop new treatments for that disease.

**10. Would you consent to the storage of hereditary material (DNA) from your blood?**

- I would give consent to the storage of DNA (1)
- I would only give consent to the storage of DNA if… (you can indicate your conditions in Question 11) (2)
- I would not give consent to the storage of DNA (3)
- Other, namely (4) ________________________________________________

Skip To:   If 10. Would you consent to the storage of hereditary material (DNA) from your blood? = I would not consent to the storage of hereditary material

**11. Under which conditions would you consent to the storage of your DNA? (You may provide more than one answer)**

- If I am kept informed of what my DNA is used for (1)
- If results (like those related to serious, hereditary disease) are communicated to me (2)
- If results (like those related to serious, hereditary disease) are not communicated to me (3)
- Depending on the purpose for which the material is used (4)

Display This Question:

If 11. Under which conditions would you consent to the storage of your DNA? (You may provide more than one answer) = Depending on the purpose for which the material is used

**11b. For what purpose(s) may your hereditary material (DNA) be used?**

- To conduct research on the genes involved in health and the emergence of disease (including rare diseases and anomalies) (1)
- To determine whether or not a hereditary disease is present (in case of concerning findings you will be informed) (2)
- In support of investigation by police/justice, only if the identity of the suspect is known *and* the suspect is missing, fugitive or deceased (3)
- Other, namely (4) ________________________________________________

Researchers use your bodily material and data for scientific research. The results of this kind of research are often shared with participants in a yearly newsletter. Sometimes it is also possible to receive a brief report with results relevant to your health. These may include: blood pressure, iron levels, etc.

**12. What kind of information pertaining to your bodily material and data would you like to receive from Sanquin? (You may provide more than one answer)**

- A yearly newsletter with general research results (does not include individual results) (1)
- An individual report with results pertaining to my health (2)
- I do not want to receive any information (3)
- Other, namely (4) ________________________________________________

Display This Question:

If 12. What kind of information pertaining to your bodily material and data would you like to receive from Sanquin? (You may provide more than one answer) = An individual report with results pertaining to my health

**12b. How would the provision of an individual report with results pertaining to your health affect your possible participation in the biobank?**

- I would be more likely to participate in biobank research if I were to receive an individual report with results (1)
- The provision of an individual report with results has no influence on my decision to participate in biobank research (2)
- I would be less likely to participate in biobank research if I were to receive an individual report with results (3)
- Other, namely (4) ________________________________________________

| Page Break |  |
| --- | --- |

The information that is collected for the purpose of a biobank could allow for the provision of advice to participants on their lifestyle with the aim of improving their health. This advice might relate to sufficient levels of physical activity or healthy eating habits.

**13. Which of the following statements apply to you?**

- I would like to receive one-time advice on improvements to my lifestyle (1)
- I am prepared to use an app that will monitor my lifestyle choices and that will send reminders and advice (2)
- I would rather not receive any lifestyle advice (3)
- Other, namely (4) ________________________________________________

Display This Question:

If 13. Which of the following statements apply to you? = I would like to receive one-time advice on improvements to my lifestyle

Or 13. Which of the following statements apply to you? = I am prepared to use an app that will monitor my lifestyle choices and that will send reminders and advice

**13b. Which of the following statements apply to you?**

- Advice on lifestyle should be limited to factors that are relevant to my donations (e.g. iron intake) (1)
- I would like to receive lifestyle advice that is relevant to my health in general, and not just to my donations (2)
- Other, namely (3) ________________________________________________

| Page Break |  |
| --- | --- |

**14. What is your biggest deterrent or concern with regard to participation in Sanquin’s biobank?**

**Thank you for filling in this questionnaire! If you have any questions or comments, please submit them below:**

End of Block: Questions about the biobank
