# Supplementary material for: Attitudes of blood and plasma donors towards biobanking for longitudinal health research
Source: PLoS One. 2025 Dec 18;20(12):e0338494. doi: 10.1371/journal.pone.0338494 (PMC12714250; doi:10.1371/journal.pone.0338494)
Supplement: Attachment — Submitted filename: Response to the reviewers.2.docx [file pone.0338494.s004.docx]

Dr. Sandar Tin Tin, Academic Editor
PLOS ONE
Amsterdam, the Netherlands, September 10^th^ 2025

Regarding: revised manuscript

Dear Dr. Sandar Tin Tin,

Thank you for your consideration of the manuscript ‘Attitudes of blood and plasma donors towards biobanking for longitudinal health research’ and supplying us with the needed improvements for the journal requirements as well as the reviewers’ comments.

***Journal Requirements:***

*1. When submitting your revision, we need you to address these additional requirements.*

*Please ensure that your manuscript meets PLOS ONE's style requirements, including those for file naming. The PLOS ONE style templates can be found at* [*https://journals.plos.org/plosone/s/file?id=wjVg/PLOSOne_formatting_sample_main_body.pdf*](https://journals.plos.org/plosone/s/file?id=wjVg/PLOSOne_formatting_sample_main_body.pdf) *and* [*https://journals.plos.org/plosone/s/file?id=ba62/PLOSOne_formatting_sample_title_authors_affiliations.pdf*](https://journals.plos.org/plosone/s/file?id=ba62/PLOSOne_formatting_sample_title_authors_affiliations.pdf)

We would like to thank the editor for this overview of PLOS ONE style requirements, with the style templates as clear and helpful examples.
We have adjusted the title page (page 1) by removing the second title (line 7, page 1), adding the asterix to the corresponding author (line 14, page 1), reordering the components in the affiliations from small to large (line 16, line 19, line 21, page 1), and replacing the address of the corresponding author by the email address and initials in parentheses (line 26 to line 29, page 1).
We also adjusted the font of the level 1 and level 2 headings throughout the manuscript, in accordance with the template of the main body (line 33 and line 52, page 2; line 94, line 95 and line 100, page 4; line 112 and line 127, page 5; line 139, line 148 and line 149, page 6; line 155, page 7; line 174, page 8; line 193, page 9; line 201 and line 212, page 10; line 275, page 12; line 277, page 13; line 329, page 15), the italics in the headings were removed (line 95 and line 100, page 4; line 112 and line 127, page 5; line 139 and line 149, page 6; line 155, page 7; line 174, page 8; line 193, page 9; and line 201, page 10), and headings were written in sentence case (line 94 and line 95, page 4; line 112, page 5; line 139, page 6).

The figure captions were made bold and not cursive (line 198-200 and line 209-211, page 10) and the table captions were made bold instead of cursive (line 154, page 6; line 163, page 7; line 192, page 9). Likewise, the supporting information titles were renamed to ‘S1 File’ and ‘S2 File’ and were made bold (line 119, page 5; line 330-332 and line 333, page 15). The empty rows to create spacing in the tables were removed (line 163, page 7; line 192, page 9). Finally, an underscore was added in the file naming of the two Supporting information files.

*2. We note that this data set consists of interview transcripts. Can you please confirm that all participants gave consent for interview transcript to be published?*

*If they DID provide consent for these transcripts to be published, please also confirm that the transcripts do not contain any potentially identifying information (or let us know if the participants consented to having their personal details published and made publicly available). We consider the following details to be identifying information:
- Names, nicknames, and initials
- Age more specific than round numbers
- GPS coordinates, physical addresses, IP addresses, email addresses
- Information in small sample sizes (e.g. 40 students from X class in X year at X university)
- Specific dates (e.g. visit dates, interview dates)
- ID numbers*

*Or, if the participants DID NOT provide consent for these transcripts to be published:
- Provide a de-identified version of the data or excerpts of interview responses
- Provide information regarding how these transcripts can be accessed by researchers who meet the criteria for access to confidential data, including:
a) the grounds for restriction
b) the name of the ethics committee, Institutional Review Board, or third-party organization that is imposing sharing restrictions on the data
c) a non-author, institutional point of contact that is able to field data access queries, in the interest of maintaining long-term data accessibility.
d) Any relevant data set names, URLs, DOIs, etc. that an independent researcher would need in order to request your minimal data set.*

*For further information on sharing data that contains sensitive participant information, please see:* [*https://journals.plos.org/plosone/s/data-availability#loc-human-research-participant-data-and-other-sensitive-data*](https://journals.plos.org/plosone/s/data-availability#loc-human-research-participant-data-and-other-sensitive-data)

*If there are ethical, legal, or third-party restrictions upon your dataset, you must provide all of the following details (*[*https://journals.plos.org/plosone/s/data-availability#loc-acceptable-data-access-restrictions*](https://journals.plos.org/plosone/s/data-availability#loc-acceptable-data-access-restrictions)*):
a) A complete description of the dataset
b) The nature of the restrictions upon the data (ethical, legal, or owned by a third party) and the reasoning behind them
c) The full name of the body imposing the restrictions upon your dataset (ethics committee, institution, data access committee, etc)
d) If the data are owned by a third party, confirmation of whether the authors received any special privileges in accessing the data that other researchers would not have
e) Direct, non-author contact information (preferably email) for the body imposing the restrictions upon the data, to which data access requests can be sent*

We express our gratitude to the editor for this point of advice, as well as the extensiveness of the included information on this topic. We think there has been a misunderstanding, as the data set does not contain interview transcripts: these are respondents’ typed responses in the free-text fields linked to the questions in the survey. Furthermore, the open answers were coded to prevent any information to be potentially identifying. With this information, could you please indicate whether anything still needs to be added regarding the consent description and how we have included the (coded) answers to the free-text fields in the dataset?

*3. We note that there is identifying data in the Supporting Information file. Due to the inclusion of these potentially identifying data, we have removed this file from your file inventory. Prior to sharing human research participant data, authors should consult with an ethics committee to ensure data are shared in accordance with participant consent and all applicable local laws.*

*Data sharing should never compromise participant privacy. It is therefore not appropriate to publicly share personally identifiable data on human research participants. The following are examples of data that should not be shared:
-Name, initials, physical address
-Ages more specific than whole numbers
-Internet protocol (IP) address
-Specific dates (birth dates, death dates, examination dates, etc.)
-Contact information such as phone number or email address
-Location data
-ID numbers that seem specific (long numbers, include initials, titled “Hospital ID”) rather than random (small numbers in numerical order)*

*Data that are not directly identifying may also be inappropriate to share, as in combination they can become identifying. For example, data collected from a small group of participants, vulnerable populations, or private groups should not be shared if they involve indirect identifiers (such as sex, ethnicity, location, etc.) that may risk the identification of study participants.*

*Additional guidance on preparing raw data for publication can be found in our Data Policy (*[*https://journals.plos.org/plosone/s/data-availability#loc-human-research-participant-data-and-other-sensitive-data*](https://journals.plos.org/plosone/s/data-availability#loc-human-research-participant-data-and-other-sensitive-data)*) and in the following article:* [*http://www.bmj.com/content/340/bmj.c181.long*](http://www.bmj.com/content/340/bmj.c181.long)*.*

*Please remove or anonymize all personal information (<specific identifying information in file to be removed>), ensure that the data shared are in accordance with participant consent, and re-upload a fully anonymized data set. Please note that spreadsheet columns with personal information must be removed and not hidden as all hidden columns will appear in the published file.*

We would like to thank the editor for her sharpness on this point of privacy, as that is very important when working with participant data. We have thoroughly investigated this point of the editor but we could not find any of the named examples of data that should not be shared in the Supporting Information file, neither any combinations of data that can become identifying. In our view the groups are also large enough to make them unidentifiable. Moreover, provinces in the Netherlands are big areas, meaning that the provinces data would not lead to identification. Nevertheless, if the locations where the respondents usually donate are the problem in this case, we could remove that specific data from the Supporting Information file. Could you please clarify which data in the Supporting Information file is considered identifying data? We would like to thank you in advance for your time.

*4. Please upload a new copy of Figures 1 and 2, as the detail is not clear. Please follow the link for more information:* [*https://blogs.plos.org/plos/2019/06/looking-good-tips-for-creating-your-plos-figures-graphics/*](https://blogs.plos.org/plos/2019/06/looking-good-tips-for-creating-your-plos-figures-graphics/)*"* [*https://blogs.plos.org/plos/2019/06/looking-good-tips-for-creating-your-plos-figures-graphics/*](https://blogs.plos.org/plos/2019/06/looking-good-tips-for-creating-your-plos-figures-graphics/)*"*

We would like to thank the editor for the provided link about clear figures. However, we do not understand the problem because they are both vector files which should have no resolution or detail issues. We could not see the problem of no clear details in our figures. We provided extra .tif versions of the figures, using the suggested Preflight Analysis and Conversion Engine (PACE) digital diagnostic tool, that we were hoping that would solve the problem. However, these .tif versions generated by the PACE tool had major problems in resolution. Therefore, we uploaded our .eps files once again, hoping that these are sufficient to meet the requirements of the figure resolution.

*5. If the reviewer comments include a recommendation to cite specific previously published works, please review and evaluate these publications to determine whether they are relevant and should be cited. There is no requirement to cite these works unless the editor has indicated otherwise.*

We would like to thank the editor for this valuable addition to the reviewers’ recommendations, which allowed us to openly look at the recommendations without feeling obliged to include them.

*6. Please review your reference list to ensure that it is complete and correct. If you have cited papers that have been retracted, please include the rationale for doing so in the manuscript text, or remove these references and replace them with relevant current references. Any changes to the reference list should be mentioned in the rebuttal letter that accompanies your revised manuscript. If you need to cite a retracted article, indicate the article’s retracted status in the References list and also include a citation and full reference for the retraction notice.*

We would like to thank the editor for this recommendation. We carefully and thoroughly checked the reference list and made some small adjustments in the references itself to make sure that the exact reference was used (line 314, line 323 and line 325, page 14). Furthermore, 2 citations were added based on the reviewers’ suggestions (line 281-283, page 13; line 319-320, page 14). In accordance, the reference numbers in the square brackets were adjusted through the whole text.
Additionally, the mentioning of a study in the text was adjusted from research/study ‘by author name et al. (year)’ to ‘from country’ (line 242 and line 250, p11).

***Reviewers' comments:***

***Reviewer's Responses to Questions***

***Comments to the Author***

*1. Is the manuscript technically sound, and do the data support the conclusions?

The manuscript must describe a technically sound piece of scientific research with data that supports the conclusions. Experiments must have been conducted rigorously, with appropriate controls, replication, and sample sizes. The conclusions must be drawn appropriately based on the data presented.*

*Reviewer #1: Yes*

*Reviewer #2: Yes*

*2. Has the statistical analysis been performed appropriately and rigorously?*

*Reviewer #1: Yes*

*Reviewer #2: No*

*3. Have the authors made all data underlying the findings in their manuscript fully available?

The* [*PLOS Data policy*](https://emea01.safelinks.protection.outlook.com/?url=http%3A%2F%2Ftrack.editorialmanager.com%2FCL0%2Fhttp%3A%252F%252Fwww.plosone.org%252Fstatic%252Fpolicies.action%2523sharing%2F1%2F010f019881cddff1-b6248ffe-7361-4c04-96df-c7f9da11cbd9-000000%2FKshm5xHjQgXm1HymJ0HnEVF7zA48yQrQVZBny4mGoeg%3D223&data=05%7C02%7C%7C70fccc05206d454badba08ddd5447265%7C84df9e7fe9f640afb435aaaaaaaaaaaa%7C1%7C0%7C638901212130370883%7CUnknown%7CTWFpbGZsb3d8eyJFbXB0eU1hcGkiOnRydWUsIlYiOiIwLjAuMDAwMCIsIlAiOiJXaW4zMiIsIkFOIjoiTWFpbCIsIldUIjoyfQ%3D%3D%7C0%7C%7C%7C&sdata=8Z2K7SOCRWCl0zbGIpTDcPMpon7WW0qahOPNZWllqKE%3D&reserved=0) *requires authors to make all data underlying the findings described in their manuscript fully available without restriction, with rare exception (please refer to the Data Availability Statement in the manuscript PDF file). The data should be provided as part of the manuscript or its supporting information, or deposited to a public repository. For example, in addition to summary statistics, the data points behind means, medians and variance measures should be available. If there are restrictions on publicly sharing data—e.g. participant privacy or use of data from a third party—those must be specified.*

*Reviewer #1: Yes*

*Reviewer #2: Yes*

*4. Is the manuscript presented in an intelligible fashion and written in standard English?

PLOS ONE does not copyedit accepted manuscripts, so the language in submitted articles must be clear, correct, and unambiguous. Any typographical or grammatical errors should be corrected at revision, so please note any specific errors here.*

*Reviewer #1: Yes*

*Reviewer #2: Yes*

*5. Review Comments to the Author

Please use the space provided to explain your answers to the questions above. You may also include additional comments for the author, including concerns about dual publication, research ethics, or publication ethics. (Please upload your review as an attachment if it exceeds 20,000 characters)*

***Reviewer #1:*** *The manuscript by Joosten and co-workers describes attitudes of blood donors towards biobanking in the Netherlands. Blood donor population has been suggested, and proved as well, to be an excellent source of biosamples from (relatively) healthy individuals. They find that a vast majority of donors are willing to consent to biobanking. The study is interesting and important addition to the current knowledge. There are a few issues I hope the authors would consider:

The survey was announced in the donor new letter and carried out July-August. Table 1 shows the basic demographics of the 409 participants. How well this population represents the overall donor population? They could show the figures of the overall donor population for comparison (perhaps no statistical comparison needed as the populations sizes are so different). I would assume that the participants were older, frequent donors who are willing to help in many different ways. There is a short discussion on the topic but the authors might raise this issue more as a possible bias.*

We would sincerely like to thank Jukka Partanen for the review and the provided suggestions.

The suggestion to dive deeper in the comparison of this population with the overall donor population has been seriously considered. We think that Jukka Partanen is right in her assumption that people who respond to the survey are different from the general donor population. But at the same time, it is difficult to prove this, because there is little published data available on the precise characteristics of the general donor population in the Netherlands. At this point, the only comparison we can make is between the mean age of men and women of the active donor population in the Netherlands in 2009, from the research by Timmer et al. (2019), and the age categories we have in our survey [4]. This suggests that the mean age of the survey population is slightly older than the mean age of the donor population. We added this to the discussion, with a note that this may indeed be a source of bias (line 260-262, page 12).

*It is interesting, or a bit surprising, that the majority of the donors did not know what biobanking means but still would be willing to give samples and data. The authors could discuss is it so that the trust to blood bank (here the Sanquin) is so strong that basically the (frequent, committed) donors would be willing to almost anything asked?*

The suggestion to discuss the fact that the majority of donors did not know what biobanking was, but still would be willing to give samples and data was very much appreciated. That is absolutely an interesting point to discuss. We have added this discussion point in line 223-226, page 10 and 11 and suggested that future research could give clearance on this subject.

*In the discussion, lines 250-251 the authors state that the sample of 400 was estimated a priori to be large enough for meaningfull analyses. They should describe in e.g. Method how this was estimated.*

The description of how the sample size calculation was performed, was added to the Materials and methods section (line 102-105, page 4).

*Minor points:
They could cite Mitchell, R. Blood banks biobanks and the ethics of donation. Transfusion (Paris) 50, 1866–1899 (2010), an editorial in which use of donor samples in biobanks was discussed.*

The suggestion to cite the research of Mitchell was very much appreciated. This was in our opinion a valuable addition to the discussion in line 235, page 11.

*Tthey might add some citations of large-scale biobank studies, such as the FinnGen (Kurki, M. I. et al. FinnGen provides genetic insights from a well-phenotyped isolated population. Nature 613, 508–519, 2023), in which biobanked blood donor samples have been used.*

Also, the suggestion to add a citation of the large biobank study of FinnGen was appreciated. We added this citation to the introduction (line 63-65, page 3), as it was a very clear example of how a study that combines blood samples and health data can be of major importance to improve our understanding of public health and disease development.

*An asset of samples from healthy individuals in disease-genetics studies is that, as being from healthy individuals, the disease progession or treatments do not confound the effects of genetic variants of possible interest. They hence may be an interesting option for molecular studies. The authors might think about this in the Discussion.*

Finally, the suggestion of adding something about the assets of samples from healthy individuals in disease-genetic studies to the discussion, was also very helpful. We found it a very good addition to the introduction instead of the discussion as it was another, clear, reason why healthy participant biobanks are needed over patient biobanks (line 59-60, page 3).

***Reviewer #2:*** *More information is needed about participant selection. The authors stated in the methodology that the survey invitation was sent to a randomly selected group. How was this done? Did the study use a sample selection software?*

We would sincerely like to thank Reviewer #2 for the review and the provided suggestions.

We appreciate the suggestion to add more information about the sample selection software that was used for the participant selection. For this, the marketing automation platform Copernica was used. Copernica offers the option to make a random selection (within a selection of e.g. donors) within the mailing list of newsletter subscribers. We added information about the use of Copernica to the Materials and methods section in line 107 and 108, page 4.

*Data analysis limited to percentages is insufficient. A more in-depth analysis involving cross-tabulations or logistic regression is required.*

We would like to thank the reviewer for the suggestion to add more in-depth analysis of the data. We definitely agree with the reviewer that it would be interesting to use cross-tabulations or logistic regression analyses to gain more insight into attitudes of blood donors towards biobanking. Looking at our dataset and the previously published literature on this topic, we are however not convinced that we currently have a solid foundation to analyze the data more in-depth. Our research aim and the applied methods are all based on the primary goal to gain more insight into blood donors’ attitudes towards biobanking, based on a survey. We have not established any research questions, prior to the start of this research project, regarding possible research hypotheses that require cross-tabulations or logistic regressions to look into group differences or predictors. Including these hypotheses, analyses and results at this stage, would not be fitting, and could even raise questions regarding possible data-dredging. Especially since our sample size and survey methods were not tailored to answer these types of questions, and we have not established a proper foundation based on previously published literature on this topic that incorporates group differences or predictors. In order to encourage other researchers to look into these aspects, we made an addition on this topic in the discussion in line 266-268 on page 12.

Thank you for your time and help.

Sincerely on behalf of all authors,

Karlijn Joosten
